# Caffeine Supplementation Is Beneficial for the Pulling Performance of Indoor Tug-of-War Athletes

**DOI:** 10.3390/biology14040354

**Published:** 2025-03-28

**Authors:** Chuan-Pao Lin, Ting-Ting Lee, Tzai-Li Li

**Affiliations:** 1 Graduate Institute of Coaching Science, National Taiwan Sport University, Taoyuan City 333325, Taiwan; chuanpao8010@gmail.com; 2 Department of Aquatic Sports, University of Taipei, Taipei City 111036, Taiwan; tingtingleeee@gmail.com; 3 Department of Sport Promotion, National Taiwan Sport University, Taoyuan City 333325, Taiwan

**Keywords:** nutritional supplementation, exercise fatigue, tug-of-war, effect size

## Abstract

This study investigated the effects of caffeine supplementation, carbohydrate mouth rinsing, and combined caffeine supplementation with carbohydrate mouth rinsing on the pulling performance and ratings of perceived exertion in indoor tug-of-war athletes. Eighteen tug-of-war athletes were recruited as participants. They underwent four supplementation protocols in a double-blind, single-factor, repeated-measures design: caffeine supplementation (CAF), carbohydrate solution mouth rinsing (CHO), combined caffeine supplementation with carbohydrate mouth rinsing (CAF-CHO), and a placebo (PLA). Each participant performed a maximal pulling test at 80% of their maximum pulling force, and the duration was recorded. Ratings of perceived exertion were also assessed. Data were analyzed using SPSS version 25.0 for Windows, and effect sizes (ES) were calculated. The main findings were as follows: (1) The CAF-CHO group showed a significantly longer pulling duration than the PLA and CHO groups (165.0 ± 69.2 s vs. 137.4 ± 48.9 s vs. 135.6 ± 66.6 s). (2) The CAF group also demonstrated a significantly longer pulling duration than the CHO group (162.0 ± 14.1 s vs. 135.6 ± 66.6 s). (3) Both the CAF-CHO and CAF groups exhibited large effect sizes (ω^2^ > 0.14) compared to the PLA and CHO groups. Both caffeine supplementation combined with carbohydrate mouth rinsing and caffeine supplementation alone can significantly enhance the duration of 80% maximal pulling performance in indoor tug-of-war athletes, with the primary effect attributed to caffeine. These results provide practical strategies for coaches and athletes to suppress fatigue and improve pulling performance during competitions or training sessions for indoor tug-of-war.

## Simple Summary

This study investigated the effects of different nutritional supplementation methods on the performance of tug-of-war athletes, with a particular focus on personal maximum pulling force and endurance time at 80% of maximum pulling force. While caffeine supplementation demonstrated effectiveness, carbohydrate mouth rinsing did not yield significant benefits in this cohort of athletes. This research contributes to understanding how nutritional strategies can enhance performance in tug-of-war.

## 1. Introduction

Tug-of-war is a sport demanding maximum strength and continuous resistance against the opponent. From the start of the competition, both sides must exert their utmost effort to pull the other side towards themselves until a winner is determined. Even though initial pulling force may be substantial, a lack of sustained pulling ability could result in defeat by the opponent. Therefore, tug-of-war athletes primarily require maintaining short-term maximal force output rather than endurance for low-intensity loads over more extended periods [1]. From the energy system perspective, tug-of-war relies on the anaerobic lactic system as the primary energy source at the start of the competition, transitioning to an aerobic–anaerobic glycolytic system during the offensive and defensive phases until both sides reach exhaustion [1]. In competitive sports, the primary goal of coaches and athletes is to enhance athletic performance through various means. This includes evaluating the specific characteristics of the sport and employing sports science-based training methods, as well as continuously seeking other strategies to improve performance [2]. In practical applications on the competition field, athletes frequently use nutritional supplements to enhance performance and increase lean body mass. The American College of Sports Medicine (ACSM) has noted that appropriate dietary supplementation can accelerate recovery from fatigue after training and improve athletic performance, making supplementation a tool for performance enhancement [2]. Recent research has further confirmed that caffeine supplementation and carbohydrate solution mouth rinsing can effectively mitigate fatigue during exercise and enhance performance [2,3,4,5,6,7,8,9,10]. However, studies explicitly applying nutritional supplements in tug-of-war have yet to be conducted.

Caffeine’s chemical structure is similar to adenosine’s, allowing it to bind to adenosine receptors. This binding reduces the interaction of adenosine with cell membrane receptors, thereby decreasing the perception of effort and feelings of fatigue during exercise [2,3]. This, in turn, promotes the metabolism of free fatty acids, conserving glycogen use. Caffeine’s chemical structure is similar to that of adenosine, allowing it to bind to adenosine receptors and decrease the interaction of adenosine with cell membrane receptors, thereby reducing perceived exertion and feelings of fatigue during exercise. Consequently, caffeine is extensively used as a supplement before or during exercise [2,3,4,5,6]. According to the guidelines and research by the International Society of Sports Nutrition (ISSN), caffeine can enhance athletic performance, including improvements in muscle strength and endurance, speed, short-distance sprinting, jumping, throwing abilities, and anaerobic performance, with the most pronounced effects observed in prolonged endurance sports. However, individual responses can vary, and caffeine supplementation might also enhance performance if applied to sports involving strength confrontation [2,4,5,6,7,8,9,10,11]. Previous research explored the effects of supplementing with 6 mg of caffeine per kilogram of body weight on jump performance and maximal muscle strength in collegiate female athletes, finding a significant increase in jump performance but no significant differences in maximal strength [12]. Another study examined the impact of caffeinated gum supplementation (300 mg caffeine, 6 g carbohydrates) on mountain bike athletes, indicating that consuming 300 mg of caffeine gum enhanced exercise performance and reduced perceived exertion [13].

Carbohydrate mouth rinsing is a method that enhances exercise performance without the need to ingest carbohydrates; it involves briefly rinsing the mouth, which stimulates sensory receptor cells in the oral cavity and influences neural pathways in the brain. When the taste receptor cells (TRCs) in the mouth are stimulated, they analyze the contacting substances; sweet stimuli from glucose, sucrose, fructose, and artificial sweeteners are received and interpreted by specific taste receptor cells, G protein-coupled receptors T1R2 and T1R3, which then release neurotransmitters to activate the sweet-taste signaling pathway. This activation stimulates regions in the brain such as the insula, frontal operculum, orbitofrontal cortex, and striatum, areas related to motor control, attention, and reward mechanisms, generating a sense of pleasure during exercise, thereby enhancing performance [7,8,9,10].

Research exploring the effects of carbohydrate mouth rinsing on enhancing athletic performance predominantly utilizes glucose or maltodextrin as components of the carbohydrate solutions [7]. Previous studies have investigated the influence of glucose and maltodextrin solutions on athletic performance, using functional magnetic resonance imaging (fMRI) to explore the regions of the brain activated by these solutions. The findings indicated that both a 6.4% glucose solution and a 6.4% maltodextrin solution significantly shortened the completion time of cyclists in time-trial events, with fMRI showing similarities in the activation patterns of the brain between glucose and maltodextrin solutions, including activation of the insula, frontal operculum, orbitofrontal cortex, and striatum [14]. Although ingesting carbohydrate solutions positively affects subsequent aerobic endurance performance, some athletes may experience gastrointestinal discomfort from consuming carbohydrates during competitions, leading to increased insulin levels, adversely affecting subsequent performance or reducing finishing levels [7]. Tug-of-war, which relies on energy supplied by the anaerobic lactic and aerobic–anaerobic glycolytic pathways, may be better suited for carbohydrate mouth rinsing to gain positive benefits, as compared to the potential gastrointestinal issues caused by ingesting liquids [8]. Carbohydrate mouth rinsing can be applied not only to athletes experiencing gastrointestinal discomfort but also to enhance the performance of athletes who cannot consume food for extended periods due to religious beliefs. Research by Bataineh et al. [15] demonstrated that carbohydrate mouth rinsing could extend the time to exhaustion and maximum treadmill speed in elite track athletes during Ramadan. Additionally, studies by Fares & Kayser [16] and Sinclair et al. [17] found that rinsing for durations between 5 and 10 s may enhance athletic performance.

While caffeine supplementation and carbohydrate mouth rinsing have been shown to enhance athletic performance and reduce fatigue, their mechanisms and pathways differ significantly. A critical question arises: Can simultaneous use of these interventions produce an additive effect on improving exercise performance and decreasing fatigue? Prior research has demonstrated that caffeine supplementation and carbohydrate mouth rinsing can significantly enhance lower limb endurance performance and reduce perceived exertion during exercise. However, they showed no significant benefits for upper limb endurance performance [18,19]. Another study investigated the effects of different supplementation methods on the repeated vertical jump test, lactate levels, and perceived exertion in male national team athletes, finding that different supplementation methods had no significant effects on short-term repeated vertical jump performance [20]. Conversely, another study concluded that, compared to the placebo group, different supplementation strategies (caffeine supplementation, carbohydrate mouth rinsing, and caffeine supplementation combined with carbohydrate mouth rinsing) significantly improved endurance in female athletes during squat, leg press, and bench press exercises. However, no significant differences were observed between the supplementation methods, and the perceived exertion (RPE) rates showed no significant differences [18]. The effects of caffeine supplementation on the pulling performance of tug-of-war athletes remain unclear. Besides needing good anaerobic and aerobic fitness, tug-of-war athletes must also employ strategies to reduce and suppress exercise-induced fatigue. This study hypothesizes that tug-of-war athletes must implement strategies to mitigate and suppress exercise-induced fatigue and maintain good anaerobic and aerobic fitness. It is proposed that if tug-of-war athletes can activate the brain’s sweet-taste central nervous system pathways, this could induce a sense of enjoyment during exercise. In that case, it may lead to a faster entry into competitive conditions, thereby effectively enhancing their performance. Therefore, this study aims to investigate the effects of caffeine supplementation, carbohydrate mouth rinsing, and the combination of both on the pulling performance and perceived exertion levels of indoor tug-of-war athletes.

## 2. Materials and Methods

### 2.1. Participants

This study recruited 18 tug-of-war athletes as participants, aged between 18 and 25 years, who had been engaged in specialized tug-of-war training for more than three years and were still actively participating in such training. Participants who did not meet any of the inclusion criteria were excluded. The researchers explained the study’s objectives and the experimental procedures, and participants became study subjects after signing an informed consent form. To standardize participants’ sensitivity to caffeine and carbohydrates, they were required to refrain from eating and consuming caffeine for at least 8 h before the experiment. Additionally, all participants were provided a standardized breakfast with identical nutritional content before the trial. The study protocol was approved by the Medical Ethical Review Committee of Fu Jen Catholic University (FJU-IRB NO C110070). The baseline characteristics of the participants are summarized in Table 1.

### 2.2. Experimental Protocol

This study employed a double-blind, single-factor, repeated-measures crossover design, with 18 participants randomly assigned to four groups, each receiving one of the following experimental treatments: caffeine supplementation (CAF), carbohydrate mouth rinsing (CHO), a combination of caffeine supplementation and carbohydrate mouth rinsing (CAF-CHO), and a placebo control group (PLA). Each experimental condition was separated by a duration of 48–72 h. Body composition analysis and personal maximum pulling force tests were conducted before the experiments. When the first and second weight measurements differed, the heavier weight was designated as the participant’s maximum pulling weight. On the day of each experiment, participants were required to consume a standardized diet and undergo supplementation one hour before the 80% maximum pulling force endurance test. Immediately following the test, participants’ perceived exertion levels were assessed using the Rating of Perceived Exertion (RPE) scale [2,18].

### 2.3. Standardized Diet

Participants were required to fast for at least 8 h before the experiment and consume a standardized nutritional breakfast provided by the researchers one hour before the experimental session. This breakfast contained approximately 433 calories, consisting of a rice ball with a nutrient composition of 36.1 g carbohydrates, 6.1 g fat, and 5.3 g protein and low-sugar, high-fiber soy milk with a nutrient composition of 23.0 g carbohydrates, 8.6 g fat, 15.3 g protein, 11.2 g sugar, and 9.0 g dietary fiber. This dietary protocol was implemented to standardize participants’ carbohydrate sensitivity [2].

### 2.4. Assessment Procedures

#### 2.4.1. Body Composition

A body composition analyzer (Jawon Medical, IOI-353, Seongnam-si, Republic of Korea) was used for the analysis. Before testing, the areas of the body in contact with the electrodes were cleaned using 75% alcohol to ensure measurement accuracy. Participants were instructed to wear loose-fitting clothing and to refrain from carrying any metal or conductive items. They were required to stand barefoot on the device while naturally gripping the handrails for the analysis [21].

#### 2.4.2. Exercise Performance Testing

##### Personal Maximum Pulling Force

The personal maximum pulling force test results were the basis for determining the intensity of the 80% maximum pulling force endurance test. The test was conducted using a personal tug-of-war machine (Convergent-Force Tug-of-War Training Machine, VETEC INDUSTRIES Inc., Taichung City, Taiwan), where participants were instructed to execute a “backward step” attacking technique, pulling a weight up to a height of 2 m before returning it to its original position. If the participant could not lift the weight to 2 m, the weight was reduced by 2.5 to 10 kg. Conversely, if the participant successfully lifted it to 2 m, the weight was incrementally increased by 2.5 to 10 kg until the participant could no longer reach the specified height. A resting interval of 3 min was enforced between each test [21].

##### 80% Maximum Pulling Force Endurance Time

The intensity for the 80% maximum pulling force endurance test was set at 80% of the results from the personal maximum pulling force test. The test used a personal tug-of-war machine (Convergent-Force Tug-of-War Training Machine, VETEC INDUSTRIES Inc., Taichung City, Taiwan). Two athletes raised the weight to the specified height of 2 m before departing. The timer was initiated as the participant stood on the outdoor tug-of-war platform. The participant was required to maintain the “tug-of-war specialized movement” until exhaustion, at which point the weight fell to the ground, and the timer was stopped [21,22].

#### 2.4.3. Supplementation

The CAF group ingested a 6.5 mg/kg caffeine capsule one hour before the test (with caffeine allocated according to each participant’s body weight; if participants consumed more than 400 mg daily, this amount was capped at 400 mg). They rinsed with a placebo (tap water) for 10 s before the test [18]. The CHO group ingested a placebo capsule (corn starch) one hour before the test and rinsed with a 100 mL solution containing 6 g of maltodextrin for 10 s before the test [18]. The CAF-CHO group ingested a 6.5 mg/kg caffeine capsule one hour before the test and rinsed with a 100 mL solution containing 6 g of maltodextrin for 10 s before the test [18]. The PLA group ingested a placebo capsule one hour before the test and rinsed with a placebo (corn starch) solution (tap water) for 10 s before the test [18].

#### 2.4.4. Rating of Perceived Exertion

The Borg [18] 10-point Rating of Perceived Exertion (RPE) scale was utilized, and participants completed the perceived exertion assessment after the pulling performance test [23].

#### 2.4.5. Data Processing

Data in this study are presented as mean ± standard deviation (Mean ± SD) and were statistically analyzed using SPSS version 25.0 for Windows. A double-masked, single-factor, repeated-measures ANOVA was conducted for statistical testing, and effect sizes (ω^2^) were calculated to verify the supplementation effects. An ω^2^ < 0.01 indicates a trivial effect, ω^2^ of 0.01 to 0.06 indicates a small effect, ω^2^ of 0.06 to 0.14 indicates a moderate effect, and ω^2^ > 0.14 indicates a significant effect [24]. The statistical significance level was set at α = 0.05.

## 3. Results

Due to three male participants experiencing discomfort during the 80% Maximum Pulling Force Endurance Time test and subsequently withdrawing from the study, the statistical analysis was conducted with a total of seven male and eight female tug-of-war athletes, comprising 15 participants. One-way repeated-measures ANOVA revealed the effects of different supplementation methods on the endurance duration (seconds) at 80% of maximum pulling force in male tug-of-war athletes (Table 2). The CAF group significantly outperformed the PLA group (173.4 ± 50.2 vs. 113.5 ± 37.0, *p* < 0.05). Additionally, the CAF group exhibited significantly better performance compared to the CAF-CHO group (173.4 ± 50.2 vs. 134.8 ± 48.8, *p* < 0.05) and the CHO group (173.4 ± 50.2 vs. 131.1 ± 15.6, *p* < 0.05) (Table 3). In female tug-of-war athletes, the CAF-CHO group demonstrated significantly higher pulling performance compared to the CHO group (191.5 ± 76.3 vs. 139.6 ± 44.0, *p* < 0.05) (Table 4). For all participants, the results indicated that the CAF group outperformed the CHO group (162.0 ± 14.1 vs. 135.6 ± 66.6, *p* < 0.05). The CAF-CHO group also exhibited significantly better performance than the PLA group (165.0 ± 69.2 vs. 137.4 ± 48.9, *p* < 0.05) and the CHO group (165.0 ± 69.2 vs. 135.6 ± 66.6, *p* < 0.05) (Table 5).

**Figure 1:**
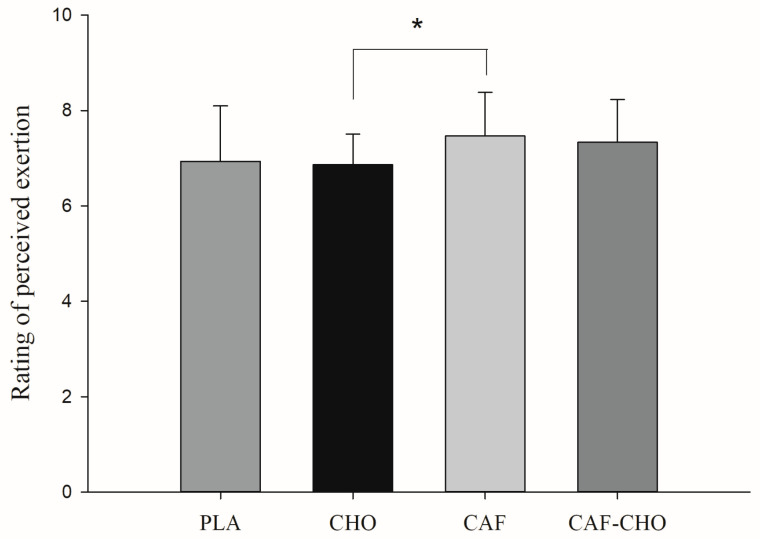
Effects of different supplementation methods on the rating of perceived exertion of Tug-of-War athletes. * Significant difference between groups.

From the perspective of effect size analysis, the CAF group exhibited a large effect size compared to the PLA group and a large effect size compared to the CHO group (ω^2^ > 0.14). Additionally, the CAF-CHO group showed a significant effect size compared to the PLA group and also displayed a large effect size when compared to the CHO group (ω^2^ > 0.14) (Table 6). Using one-way repeated-measures ANOVA to analyze the effects of different supplementation methods on the perceived exertion levels of all tug-of-war athletes (seven males and eight females), the results indicated a significant difference between the CAF group and the CHO group (7.4 ± 0.9 vs. 6.8 ± 0.6). In contrast, no significant differences were observed among the other groups (Figure 1).

## 4. Discussion

This study is one of the few practical application research projects focusing on nutritional supplementation in the context of tug-of-war. The main findings of this research are as follows: (1) The pulling performance of the CAF-CHO group was significantly higher than that of the PLA group and the CHO group (165.0 ± 69.2 s vs. 137.4 ± 48.9 s vs. 135.6 ± 66.6 s); (2) The pulling performance of the CAF group was significantly higher than that of the CHO group (162.0 ± 14.1 s vs. 135.6 ± 66.6 s); (3) The CAF-CHO group exhibited a large effect size compared to the PLA group and the CHO group (ω^2^ > 0.14), and the CAF group also demonstrated a large effect size compared to the PLA group and the CHO group (ω^2^ > 0.14).

The other results of this study indicate that the CAF-CHO group demonstrated a significantly longer duration of pulling performance at 80% of maximum pulling force compared to the PLA group and the CHO group. Additionally, the CAF group showed a significantly longer pulling duration than the CHO group; however, no significant differences were observed between the CAF-CHO group and the CAF group, nor between the CHO group and the PLA group. These findings are consistent with previous research, which explored the effects of caffeine supplementation, carbohydrate mouth rinsing, and combining both on the number of repetitions in a 10 RM resistance training setting. The study found that all four supplementation methods increased the number of repetitions in female athletes during the 10 RM resistance training; however, the combination of caffeine supplementation and carbohydrate mouth rinsing (CAF-CHO group) did not produce the expected additive effect [18]. Another study investigated the differences in repeated vertical jump tests, lactate levels, and perceived exertion among national team male athletes using different supplementation methods (CAF group, CHO group, and CAF-CHO group). The results indicated no significant differences in jumping performance, lactate levels, or perceived exertion among the supplementation methods, with none of the individual or combined supplementation methods significantly enhancing the repeated vertical jump performance of the national team male athletes [20]. This study concludes that the CAF-CHO and CAF groups had enhanced benefits in the duration of the pulling performance at 80% of maximum pulling force, primarily attributed to the effects of caffeine supplementation. The influence of caffeine includes enhancing muscle activation through stimulation of the central nervous system and reducing perceived exertion and fatigue during exercise due to its antagonistic effects [2]. These findings confirm the positive effects of caffeine supplementation on the pulling performance of indoor tug-of-war athletes.

The results of this study indicated no significant difference in pulling performance between the CHO group and the PLA group. A review of previous research investigating the effects of carbohydrate mouth rinsing on athletic performance shows that most results indicated a positive impact. However, factors such as the fasting duration before rinsing, the rinsing duration, and the composition of the carbohydrate solution may influence the receptor response in the oral cavity, indirectly affecting performance. Furthermore, results may vary under different exercise environmental conditions and across various types of exercise. Prior studies have examined the effects of rinsing with varying concentrations of carbohydrate solutions (8%, 6%, 4%, and 0%) on one-hour cycling time-trial performance in a non-fasted state. The findings revealed no significant differences in completion times, power output, or physiological indicators (such as blood glucose and lactate levels) across the four supplementation methods. This may be attributed to the body’s low demand for carbohydrates, which likely hindered improvements in subsequent athletic performance [25]. Another study utilizing a 6.4% maltodextrin solution compared with a placebo solution examined the effects on 30 min cycling sprints at 90% of peak ventilation threshold after food intake, after a 12 h fast, and after 12 h of glycogen depletion. The results indicated that the maltodextrin rinsing group demonstrated better sprint performance after the 12 h fast and after glycogen depletion than the fed state [26]. A previous literature review summarizing the impact of carbohydrate mouth rinsing on athletic performance suggested that fasting for over 2 h before exercise, engaging in 60–90 min of moderate- to high-intensity cycling or running, and rinsing with a 6–8% glucose solution or a 6.4–10% maltodextrin solution for 10 s before and during exercise may enhance endurance performance [10]. This study hypothesizes that the lack of significant difference in the 80% maximum pulling force endurance time between the CHO and the PLA groups may be due to participants eating one hour before the experiment, reducing the body’s demand for carbohydrates. Without carbohydrate deficiency, the activation of the brain’s sweet-taste central nervous system pathways may have been diminished or not fully engaged, leading to a lack of enhancement in the sense of enjoyment during exercise and consequently inhibiting improvements in athletic performance.

This study indicated no significant difference between the CAF and PLA groups regarding the 80% maximum pulling force endurance time. The placebo effect originated during World War II when a U.S. Army anesthesiologist, facing a shortage of analgesic morphine, temporarily used saline solution as a substitute, resulting in over a third of soldiers experiencing similar pain relief effects, thus known as the placebo effect [27]. Previous studies examined the effects of rinsing with a 6% carbohydrate solution, a placebo solution, and no rinsing on 10 km cycling time-trial performance during fasting days (fasting for at least 12 h). The results showed that rinsing with either the carbohydrate or placebo solution during the warm-up phase significantly outperformed the no-rinse condition regarding completion time and power output. Contrary to initial expectations, the placebo solution rinsing also considerably enhanced performance compared to no rinsing, seemingly related to the placebo effect and the prolonged dehydration experienced by the participants [28]. This study posits that the lack of significant difference in the 80% maximum pulling force endurance time between the CAF group and the PLA group may be attributed to the placebo effect in some participants, thereby influencing the variability of the research findings.

To standardize the impact of diet on participants during testing, this study provided breakfast with identical nutritional content to the subjects before the experiment. However, since participants had already consumed carbohydrates before the test, they may not have been deficient in energy derived from carbohydrates during the subsequent short-duration exercise. Consequently, the effects of carbohydrate mouth rinsing in this study may not have been significant. Future studies are recommended to simulate competitive tug-of-war scenarios to investigate the effects of carbohydrate mouth rinsing on the pulling performance of athletes. This study confirms the beneficial effects of caffeine supplementation on the pulling performance of tug-of-war athletes, suggesting that coaches and athletes consider the findings as methods and strategies to mitigate exercise fatigue and enhance pulling performance during competitions and training sessions.

## 5. Conclusions

Caffeine supplementation combined with carbohydrate mouth rinsing and caffeine supplementation alone significantly enhanced the specific athletic performance of tug-of-war athletes in the 80% maximum pulling force endurance test, with the primary effect likely stemming from caffeine. The lack of significant benefits from carbohydrate mouth rinsing may be attributed to the participants’ low sensitivity to carbohydrate demand, which limited the activation of the neural pathways associated with the brain’s sweet-taste central system. Further investigation is needed to explore the effects of carbohydrate mouth rinsing on the pulling performance of tug-of-war athletes.

## Figures and Tables

**Table 1:** Characteristics of participants.

Parameters	Overall (n = 18)	Female (n = 8)	Male (n = 10)
Age (years)	20.17 ± 2.07	21.50 ± 2.45	19.10 ± 0.74
Height (cm)	170.06 ± 8.14	162.38 ± 5.13	176.20 ± 3.22
Weight (kg)	73.17 ± 8.56	68.99 ± 9.38	76.51 ± 6.49
Skeletal Muscle Mass (kg)	51.84 ± 6.86	45.39 ± 4.38	57.00 ± 2.71
Fat-Free Mass (kg)	56.07 ± 7.24	49.36 ± 4.84	61.43 ± 3.00
Fat Mass (kg)	17.10 ± 5.28	19.63 ± 5.65	15.08 ± 4.20
Body Fat Percentage (%)	23.28 ± 6.11	28.10 ± 4.52	19.43 ± 4.17
Maximum Pulling Force (kg)	136.4 ± 13.1	135.3 ± 14.9	137.4 ± 11.4

**Table 2:** Effects of different supplementation methods on 80% maximum pulling force endurance time in tug-of-war athletes (seconds).

	PLA	CAF	CHO	CAF-CHO
Male (n = 7)	113.5 ± 37.0	173.4 ± 50.2 *	131.1 ± 15.6 ^a^	134.8 ± 48.8 ^a^
Female (n = 8)	158.3 ± 50.4	152.0 ± 59.8	139.6 ± 44.0	191.5 ± 76.3 ^b^
Overall (n = 15)	137.4 ± 48.9	162.0 ± 14.1	135.6 ± 66.6 ^a^	165.0 ± 69.2 *^,b^

* Significant difference from the PLA group; ^a^ Significant difference from the CAF group; ^b^ Significant difference from the CHO group. *p* < 0.05.

**Table 3:** The effects of different supplementation methods on the pulling performance of male tug-of-war athletes (*p*-values).

Group (n = 7)	PLA (113.5 ± 37.0)	CHO (131.1 ± 15.6)	CAF (173.4 ± 50.2)	CAF-CHO (134.8 ± 48.8)
PLA	N/A	<0.174	<0.012 *	<0.088
CHO	<0.174	N/A	<0.035 *	<0.818
CAF	<0.012 *	<0.035 *	N/A	<0.005 *
CAF-CHO	<0.088	<0.818	<0.005 *	N/A

* Significant difference between groups. *p* < 0.05. N/A: Not Applicable.

**Table 4:** The effects of different supplementation methods on the pulling performance of female tug-of-war athletes (*p*-values).

Group (n = 8)	PLA (158.3 ± 50.4)	CHO (139.6 ± 44.0)	CAF (152.0 ± 59.8)	CAF-CHO (191.5 ± 76.3)
PLA	N/A	<0.061	<0.531	<0.115
CHO	<0.061	N/A	<0.363	<0.008 *
CAF	<0.531	<0.363	N/A	<0.123
CAF-CHO	<0.115	<0.008 *	<0.123	N/A

* Significant difference between groups. *p* < 0.05. N/A: Not Applicable.

**Table 5:** The effects of different supplementation methods on the pulling performance of overall tug-of-war athletes (*p*-values).

Group (n = 15)	PLA (137.4 ± 48.9)	CHO (135.6 ± 66.6)	CAF (162.0 ± 14.1)	CAF-CHO (165.0 ± 69.2)
PLA	N/A	<0.831	<0.072	<0.022 *
CHO	<0.831	N/A	<0.024 *	<0.027 *
CAF	<0.072	<0.024 *	N/A	<0.852
CAF-CHO	<0.022 *	<0.027 *	<0.852	N/A

* Significant difference between groups. *p* < 0.05. N/A: Not Applicable.

**Table 6:** Effects of different supplementation methods on 80% maximum pulling force endurance time in tug-of-war athletes.

Group/ES (n = 15)	PLA (137.4 ± 48.9)	CHO (135.6 ± 66.6)	CAF (162.0 ± 14.1)	CAF-CHO (165.0 ± 69.2)
PLA	0	0.030	0.683	0.460
CHO	0.030	0	0.548	0.432
CAF	0.683	0.548	0	0.060
CAF-CHO	0.460	0.432	0.060	0

ES: Effect size. *p* < 0.05.

## Data Availability

The data presented in this study are available upon request from the corresponding author.
